# Factor Structure of the Korean Version of the Occupational Socialization of Beginning Physical Education Teachers Scale in the Context of the COVID-19 Pandemic and Its Relationship with Health Perception Education

**DOI:** 10.3390/ijerph19127069

**Published:** 2022-06-09

**Authors:** Seung-Man Lee, Eui-Jae Lee, Wi-Young So, Jong-Hyuck Kim

**Affiliations:** 1 Department of Physical Education, College of Education, Korea University, Seoul 02841, Korea; lsm14pe@korea.ac.kr; 2 Department of Physical Education, Graduate School of Education, Sogang University, Seoul 04107, Korea; freedom_jae@hanmail.net; 3 Sport Medicine Major, College of Humanities and Arts, Korea National University of Transportation, Chungju-si 27469, Korea; 4 Department of Medical Beauty Care, Jungwon University, Goesan-gun 28024, Korea

**Keywords:** beginning physical education teachers, COVID-19 pandemic, factor structure, health perception education, occupational socialization

## Abstract

This study aimed to explore the factor structure of the Korean version of the occupational socialization of beginning physical education teachers scale, in the context of the COVID-19 pandemic, and to verify its relationship with health perception education. In October 2021, 257 Korean beginning physical education teachers were enrolled in this study. Data were analyzed using frequency analysis, exploratory factor analysis, confirmatory factor analysis, descriptive statistics, reliability analysis, and multiple regression analysis. Regarding the findings, first, the occupational socialization of beginning physical education teachers scale showed a six-factor structure: role recognition, past physical education class experience, pre-service teacher education, organizational atmosphere, fellow physical education teachers, and sports facility. Second, occupational socialization of beginning physical education teachers showed a partial positive effect on health perception education. These results suggest that the Korean Metropolitan and Provincial Offices of Education and Korean schools should develop various methods to support and ensure the occupational socialization of beginning physical education teachers. Such efforts may enable these new professionals to effectively adapt to their schools, teaching roles, and provide effective health education to students under the difficult context of the COVID-19 pandemic, wherein normal educational activities are hindered.

## 1. Introduction

Beginning teachers can be defined as those who are taking their first steps in the teaching role and incorporating the social responsibility that befits the teaching profession. Even after completing a rigorous process and learning how to perform their jobs, many teachers continue to struggle with the various roles associated with this profession. Furthermore, within the realm of physical education, the school site is often an unfamiliar place for beginning physical education teachers. Adding to this unfamiliarity, beginning teachers must undergo a period of trial and error to become skilled teachers, and there is no learning period to facilitate their adaptation to the job. On the contrary, they are often required to meet students, teach classes, and carry out their work as soon as they enter the teaching profession. Research shows that beginning teachers often experience various difficulties during these processes [1].

Currently, the COVID-19 pandemic is also an aggravator, which transformed schools into chaotic environments. For example, in 2020, the Korean government decided to postpone the start of school classes for the first time in its history, with education continuing in a mixed method that combined online and face-to-face classes [2]. Furthermore, face-to-face communication has been limited during the COVID-19 pandemic, leading teachers with many years of experience to face difficulties when attempting to convey various school life perks and know-how to beginning teachers. Previous studies have reported the difficulties of teaching physical education classes with the COVID-19 situation [3,4,5]. Considering the importance of occupational socialization for beginning physical education teachers [6], we see space for more in-depth discussions regarding this topic.

Teacher occupational socialization refers to the process by which teachers enter the school environment, develop their perspectives about the school culture, environment, and interactions with fellow teachers, and adapt to the school by internalizing various knowledge, behaviors, norms, values, and beliefs [7,8]. Studies on teacher occupational socialization in physical education have generally analyzed workplace socialization or the “adaptation process to become a teacher”, focusing on how to learn the knowledge, skills, and attitudes necessary to become an effective member of this profession [9]. Occupational socialization is the main topic of socialization after adulthood, and studies on occupational socialization have been reported in various occupational groups [10,11,12].

Developed countries emphasize that prevention is more important than treatment, and management is more important than prevention, making it an important task to lay the foundations for people to engage in lifelong health care and disease prevention; this is operationalized by emphasizing strategies that incentivize people to acquire healthy habits during childhood [6]. This process can be summarized into the concept of health perception, which is defined as a subjective process of becoming conscious of external stimuli related to health through our senses [13,14]. Meanwhile, health perception education refers to the systematic guidance of health awareness in schools, and students should receive classes on this subject [15]. Additionally, the world’s unpreparedness for the COVID-19 pandemic brought the topic of health and health awareness to the forefront of the values that must be taught in school. Notably, beginning physical education teachers are required to teach an important health curriculum during the COVID-19 situation. As such, it is likely that the factors of occupational socialization experienced by first-time teachers during the COVID-19 pandemic situation are closely related to health awareness education.

Despite the current relevance of the subject of health, to the best of our knowledge, there are no studies on the relationship between occupational socialization of beginning physical education teachers and health perception education, nor are there any on this topic in the context of the COVID-19 pandemic. Together, it is necessary to examine the effects of occupational socialization on the will of teachers who instruct teachers to have appropriate health awareness and deliver relevant instructional practice regarding this topic. This current study focused on health perception education, considering it as a variable that could affect the occupational socialization of beginning physical education teachers within the context of the COVID-19 pandemic.

In the COVID-19 context, beginning physical education teachers have been put in a situation where they have to teach students at school without preparing for student lessons. In general, beginning physical education teachers receive help from experienced teachers on practical methods of guiding students, adaptation to school culture, and administrative work. In the study of Deglau and O’Sullivan [16], it is argued that multiple trainings are an effective way to develop teacher professionalism. Accordingly, previous studies have argued that it is an important opportunity for teachers to develop with each other through the teacher community [17,18,19]. However, in a situation where interpersonal contact is not recommended in the context of COVID-19, starting teachers face various difficulties in a situation where it is difficult to receive help from experienced teachers. Therefore, research on the occupational socialization of beginning physical education teachers is necessary in the COVID-19 context.

Despite the lack of studies on the aforementioned relationship, some researchers have examined the importance, process, and countermeasures of occupational socialization among beginning physical education teachers. Regarding the stages of growth and retirement of teachers, Templin et al. [20] found that some socialization factors either promote or hinder socialization at each stage of socialization; described how the process is carried out; identified each value, perception, and attitude of teachers who are at this stage; and through which processes these values, perceptions, and attitudes develop and change. Regarding this process, Richards and Templin [21] emphasized the importance of the beginning-teacher period for physical education teachers, presenting a support program that promotes occupational socialization for beginning physical education teachers at the national level. Considering the importance of analyses on how to learn the knowledge, skills, and attitudes necessary to become an effective member of a specific profession, efforts are being made to approach the occupational socialization of beginning physical education teachers from multiple perspectives [9]. For example, Choi [3] conducted a case study on the adaptation process of beginning physical education teachers in middle schools and Kim et al. [22] explored how to adapt to or overcome the conflict between teaching and experience that is perceived by these physical education teachers at the beginning of their careers, again within the context of middle schools. In addition, Lee and Yoo [23] conducted a study to examine the growth process of beginning physical education teachers through educational practice community activities. Richards et al. [24] and Cho et al. [25] have also comprehensively analyzed studies on the occupational socialization of physical education teachers, suggesting a plan for promoting this socialization in a positive direction.

Specifically, previous studies on occupational socialization of beginning physical education teachers are reported as follows Romar and Frisk [26] noted the effect of occupational socialization on the physical education knowledge, confidence, and education of novice teachers. Curtner-Smith et al. [27] conducted a study on the effect of occupational socialization on novice teachers’ interpretation and delivery of physical education. Adamakis and Zounhia [28] observed the effect of occupational socialization on the beliefs of physical education teachers about four important educational outcomes as a cross-sectional study method, and Curtner-Smith [29] investigated occupational socialization focusing on how the First-Year Physical Education program affected the beginning physical education teachers’ perspective and practice.

In summary, most prior research on occupational socialization has provided in-depth analyses of the actual process of occupational socialization and used either qualitative or literature review designs. However, and despite the importance of occupational socialization for beginning physical education teachers, studies have not been conducted to explore the subscales of occupational socialization for these teachers and the relationships of this occupational socialization with other variables within this context. Therefore, in this study, we included occupational socialization of beginning physical education teachers as a variable to reveal its relationship with other variables; this was operationalized by exploring the factor structure of occupational socialization of beginning physical education teachers and confirming its relationship with health perception education, all in the context of the COVID-19 pandemic. Through this study, we attempted to provide basic data for guiding future research aimed at conducting in-depth analyses of the socialization of beginning physical education teachers. In particular, there is a scale for occupational socialization, but a scale for beginning physical education teachers has not been developed. Therefore, in this study, the scale developed in the study of Glatthorn [30] was modified to suit the beginning physical education teachers, and the factor structure was explored.

Therefore, this study aimed to explore the factor structure of the Korean version of the occupational socialization of beginning physical education teachers scale in the context of the COVID-19 pandemic and to verify its relationship with health perception education. The following are the research questions: (1) how are the factors of the Korean version of the occupational socialization of beginning physical education teachers scale structured in the context of the COVID-19 pandemic? (2) What effect does the occupational socialization of beginning physical education teachers have on health perception education in the context of the COVID-19 pandemic?

## 2. Methods

### 2.1. Participants

In this study, to obtain data, a convenience sampling method that allows researchers to easily obtain samples were used. Specifically, over the past three years, 1500 beginning physical education teachers have started working at schools. The researchers surveyed 257 beginning physical education teachers appointed in Seoul and Gyeonggi Province from 2019 to 2021. Study participants’ ages ranged from 25 to 30 years old. The survey was conducted online in November 2021 in compliance with the government’s social distancing measures due to the COVID-19 pandemic. The demographic characteristics of participants are shown in Table 1. This study was conducted after obtaining approval from the Institutional Review Board of KyungHee University (KHGIRB-20-145) and the participants of this study voluntarily signed the written informed consent.

### 2.2. Instruments

Data were collected through a survey application. Regarding questionnaire items, we chose tools that have been tested for reliability and validity in previous studies and which were judged by the research team to be consistent with the study aims. Specifically, the questionnaire was divided into three sections: (1) the general characteristics of study participants; (2) the occupational socialization of beginning physical education teachers; and (3) health perception education. In the first section of the questionnaire, there were three questions about sex, school level, and working years.

In previous studies, a study on the scale of occupational socialization has already been reported [30,31,32,33], but a scale for beginning physical education teachers has not been developed. Therefore, in this study, the scale developed in the study of Glatthorn [30] was modified to suit the beginning physical education teachers to explore the factor structure. In the second section of the questionnaire, we used a modified version of the scale developed by Glatthorn [30], adapting it to comply with the situations relevant to occupational socialization of beginning physical education teachers in Korea. First, the English questionnaire was translated into Korean. In addition, Delphi verification was conducted to verify the validity of the translated questionnaire. In the first preliminary survey, the results of Delphi verification were investigated in a closed type (2-point scale) of 8 experts (1 physical education professor and 7 physical education teachers with a doctorate degree), and 5 questions that did not meet the criteria were removed. In addition, since we explored the factor structure of the occupational socialization of beginning physical education teachers scale, a confirmatory factor analysis was conducted to confirm the subscales, and then a reliability analysis was conducted.

In the third section of the questionnaire, we used a modified version of the Health Perception Scale by Ware [34]—which in turn had the validity and reliability of its Korean version checked by Barakat et al. [35], Jones [36], and Lee et al. [37]—to measure health perception education; we modified it according to the needs of the current study. This scale comprises 6 subscales, including mental health management, disease management, physical activity management, sleep management, dietary management, and hygiene and health management. In this study, the Cronbach’s α coefficients for the Health Perception Scale and its subscales ranged from 0.712 to 0.934, indicating that the scale was reliable based on the parameters described by Tavakol and Dennick [38]. The scales in the second and third sections of the questionnaire were both structured as a five-point Likert scale ranging from 1 to 5 (completely agree, 5 points; agree, 4 points; neutral, 3 points; disagree, 2 points; and do not agree at all, 1 point). The obtained data were analyzed by constructing sub-factors through Item Parceling. Namely, this study verified the content validity, concentration validity, discriminant validity, and intra-item reliability.

### 2.3. Data Collection and Analysis

This study is a cross-sectional study, and data were collected through a survey in November 2021. Data were analyzed using SPSS and AMOS, version 24.0 (IBM Corporation) as follows. First, frequency analysis was performed to explore the participants’ demographic characteristics. Second, for reliability analysis, the Cronbach’s α coefficients of the measurement tools were calculated. Third, descriptive statistics were used to sub-variables of beginning physical education teachers’ occupational socialization. Fourth, exploratory factor analysis was conducted to explore the factor structure of the occupational socialization of beginning physical education teachers scale. Fifth, correlation analysis and multiple regression analysis were performed to verify the relationship between occupational socialization of beginning physical education teachers and health perception education. In the multiple regression analysis, the relationship was verified by setting the socialization of beginning physical education teachers as an independent variable and health perception education as a dependent variable. The significance level of all statistics was set to 0.05.

## 3. Results

### 3.1. Exploring the Factor Structure of the Occupational Socialization of Beginning Physical Education Teachers Scale

#### 3.1.1. Exploratory Factor Analysis

At this stage, we conducted a principal component analysis, with varimax, an orthogonal permutation method, being used to simplify factor loading. In the social sciences, factor and item variables are considered significant when the eigenvalue is 1.00 or more and the factor loading is 0.40 or more, and the variable can be considered very important when its loading exceeds 0.50 [39]. Accordingly, the selection criteria for these variables in the current study were an eigenvalue of 1.00 or higher and a factor loading of 0.40 or higher.

To analyze whether the sample size to be analyzed is suitable for factor analysis, we conducted a Kaiser–Meyer–Olkin (KMO) test. The sample adequacy measure was 0.898, which was judged to be appropriate based on Kaiser’s criteria: 0.80 or higher represents a good value [39]. Furthermore, Bartlett’s sphericity test results showed a coefficient of 3970.543 (*p* < 0.001), confirming the appropriateness to reject the null hypothesis (i.e., that the correlation matrix is 0.00) and conducting factor analysis between the variables. Moreover, the total variance explained was 69.82%.

Exploratory factor analysis led to the extraction of six factors, and the results are shown in Table 2. Based on these results, one item (item 5) was excluded, and based on the detailed contents of the six factors, the subscales were named as follows: role recognition, past physical education class experience, pre-service teacher education, organizational atmosphere, fellow physical education teacher, and sports facility.

#### 3.1.2. Confirmatory Factor Analysis

To verify questionnaire validity, confirmatory factor analysis was performed. The model fit was set at: a chi-square/degree of freedom (CMIN/DF) value of 2.00 or less; a root mean square residual (RMR) value of 0.05 or less; a goodness-of-fit-index (GFI), a normed fit index (NFI), an incremental fit index (IFI), and a comparative fit index (CFI) value of 0.90 or more; and a root mean square error of approximation (RMSEA) value of 0.07 or less [40]. The results of confirmatory factor analysis are shown in Table 3.

The initial goodness-of-fit results were found to be generally inadequate. Accordingly, items with a squared multiple correlation (SMC) value of 0.40 or less, which is an index used to determine how much the measured variable explains the latent variable [40], were repeatedly removed. As a result, 21 items were deleted, leaving a total of 6 factors and 24 items. The final fitness index for this version of the scale is shown in Table 4. Although the GFI value was slightly lower than the reference value (0.90), the CFI (0.942) was higher than the reference value (0.90). Research describes that because GFI can be affected by inconsistency due to sample characteristics, CFI, which is not affected by sample characteristics, is viewed as being more important as a goodness-of-fit criterion than GFI [41]. Furthermore, in confirmatory factor analysis, the model is different from the actual data. Thus, to verify the degree of conformity, the research describes the importance of considering both CFI and RMSEA values (not affected by sample size) over chi-square values (affected by sample size) [41]. Based on prior literature and the results, the scale was judged to have a good fit index.

#### 3.1.3. Verification of Convergent and Discriminant Validity

We conducted convergent and discriminant factor analysis for the extracted subscales. The results of confirmatory factor analysis are shown in Table 4. Regarding convergent validity, the standardized coefficient value of all questions ranged from 0.571 to 0.914, with all items showing a value of 0.50 or more [40]. In addition, the resulting average variance extracted (AVE) value ranged from 0.975 to 0.993, which was significant because it was higher than 0.70 [40].

Regarding discriminant validity, the values for the correlations between the average variance extracted and each variable are shown in Table 5. Since the square of the correlation coefficient of role recognition and past physical education class experience (the highest correlation) was 0.403, it was lower than the average variance extracted for role recognition (0.979) and past physical education class experience (0.961), implying that discriminant validity was low. It was still deemed that the scale showed appropriate discriminant validity.

### 3.2. Descriptive Statistical Analysis and Reliability of the Occupational Socialization of Beginning Physical Education Teachers Scale

The descriptive statistics and reliability values of the total scale and subscales are shown in Table 6. Mean values ranged from 3.41 to 4.35, and the standard deviation values ranged from 0.56 to 0.78. Next, skewness and kurtosis were calculated, with acceptable values for skewness being <±3.0 [42] and for kurtosis being <±10.0 [43]. These values are the basis for examining a univariate normality violation as well as for confirming the normal distribution of the data. In this study, the absolute values of skewness ranged from 0.03 to 0.61, while those for kurtosis ranged from 0.19 to 0.68; the values for both parameters satisfied the conditions for confirming data distribution normality. The Cronbach’s α coefficient of the measurement variables used to measure each potential factor is shown to be 0.774 to 0.923, which is reliable.

### 3.3. Effect of Occupational Socialization on Health Perception Education

The effects of occupational socialization of beginning physical education teachers on health perception education are shown in Table 7. First, the *t*-value for the positive effect of occupational socialization of beginning physical education teachers on health perception education was 15.892, and it was statistically significant. Hence, if the level of occupational socialization of beginning physical education teachers is high, health perception education also increases.

Furthermore, the regression model showed an F-value of 47.279 (*p* < 0.001) and explanatory power of 32.9%, with a modified R^2^ = 0.329 for the regression equation. Specifically, the subscales of role recognition (β = 0.178, *p* = 0.001), pre-service teacher education (β = 0.184, *p* < 0.001), and fellow physical education teacher (β = 0.247, *p* < 0.001) were found to have a positive effect on health perception education. Nonetheless, the subscales of past physical education class experience (β = 0.088, *p* = 0.089) and organizational atmosphere (β = 0.054, *p* = 0.305) were found to have no significant effect on health perception education.

## 4. Discussion

The purpose of this study was to verify how the occupational socialization factor structure of beginning physical education teachers is constituted and how it relates to health awareness education in the COVID-19 context. Based on the study results, comparison with previous studies, interpretation of the results, and the study implications are presented.

### 4.1. Interpretation of the Findings

First, results showed that the occupational socialization of beginning physical education teachers scale comprises six subscales: role recognition, past physical education class experience, pre-service teacher education, organizational atmosphere, fellow physical education teacher, and sports facility. In Choi’s [6] study, the occupational socialization of beginning physical education teachers scale had nine subscales, which were divided into individual and situational factors. Specifically, the individual factors were (1) personality, (2) motivation for choosing a teaching position and recognition of the role of teachers, (3) sports and physical education classes experienced in the past, and (4) pre-service teacher education; the situational factors were (1) organizational atmosphere, (2) workload and miscellaneous work, (3) community characteristics, (4) poor sports facilities and equipment, and (5) human relationships. After reviewing these scales, it becomes clear that the results of Choi’s [6] study differ from those of this study, as their scale was divided into two dimensions, whereas all our subscales were entered into a single dimension. In addition, Cho et al. [16] conducted a systematic literature analysis on the occupational socialization of beginning physical education teachers, finding that it was classified into (1) a personal dimension, (2) a professional dimension, and (3) an ecological dimension based on the perspective of Vonk [44]. As such, the factor structure derived in this study was different from the factor structure of occupational socialization of beginning physical education teachers suggested by Choi et al. [25]. These results can be seen as a result of the difference in research methodology, and Choi et al. [25] categorized the sub-factors of occupational socialization of beginning physical education teachers through qualitative research methods, but in this study, the factor structure was analyzed through questionnaire-based quantitative research.

In particular, Cho et al. [16] argued that there was a close relationship between the difficulties experienced and adaptation strategies used by beginning physical education teachers in the occupational socialization process, as well as that “colleagues” and “students” acted as key phenomena in the process. Nonetheless, Choi [6] and Cho et al. [25] conducted a qualitative and a literature review study, respectively, hence differing from the quantitative nature of this current study, and their results regarding the socialization process also differed from ours. Specifically, while their studies showed a multidimensional factor structure [6,25], ours showed a single-dimension factor structure.

Second, occupational socialization of beginning physical education teachers had a positive effect on health perception education. Specifically, role recognition, pre-service teacher education, and fellow physical education teacher subscales had a positive effect on health perception education, albeit the other three subscales did not significantly affect health perception education. The studies related to COVID-19 have been mainly reported from 2020. Corona-related studies are mainly announcing the results of studies on the reduction in physical activity [45,46,47] and preparation for the post-Corona era [48,49]. However, as described in the Introduction section of this manuscript, to our knowledge, there are no previous studies on the direct relationship between the occupational socialization of beginning physical education teachers and health perception education. Thus, we found limitations when attempting to directly compare these results with other studies.

The three subscales of occupational socialization of beginning physical education teachers (role recognition, pre-service teacher education, and fellow physical education teacher) had a positive effect on health perception education. Since beginning physical education teachers are responsible for recognizing their roles as teachers, these results for the role recognition subscale may be related to these teachers making efforts to guide students’ health values within the context of the COVID-19 pandemic. In addition, it is recognized that five areas of physical education (i.e., health, challenge, competition, expression, and safety) should be guided in a balanced manner during pre-service teacher education, and it can be seen as a result of sufficient acquisition of professional knowledge in the health area. Furthermore, the positive effect of the fellow physical education teacher subscale may be related to the possibility that teachers can share their pedagogical knowledge with peers through professional, community-based teacher learning activities, and teachers may be capable of developing more effective classes as they discuss teaching methods for health perception education with peers.

However, the subscales of past physical education class experience, organizational atmosphere, and sports facility did not affect health perception education. Regarding the non-effect of socializations on past physical education class experience, we deem it highly likely that the physical education classes, which current beginning physical education teachers experienced when they were adolescents, were mainly focused on sports functions, rather than health knowledge. This may explain why past class experience did not have a significant impact on the current situation, as the importance of and the social demand for health and hygiene topics to be explored during physical education classes increased due to the unexpected outbreak of the COVID-19 pandemic. In addition, the organizational atmosphere subscale did not likely affect health perception education, because in Korean schools, beginning physical teachers are not given roles within the context of classes and administrative work that are significantly different from the roles of experienced teachers.

### 4.2. Practical Implications of the Study

The practical meaning and implications of the results derived from this study can be presented as follows. In the context of the COVID-19 pandemic, teacher occupational socialization has been more restricted than before the pandemic, albeit it remains necessary for these teachers to be able to adapt more quickly to their jobs and guide students more skillfully. Accordingly, many researchers have explored the topic and provided suggestions for effectively promoting the occupational socialization of beginning physical education teachers. First, the studies by Deglau and O’Sullivan [16], O’Sullivan [17], Oh [18], Cho [19], and Cho et al. [25] argue that community-based teacher learning activities should be encouraged and supported. Specifically, one study remarks that community-based activities may facilitate communication between teachers and the creation of an environment that enables sharing experiences among professionals, so that such activities may serve as a cornerstone for the growth of all teachers [50]. For beginning physical education teachers to adapt well to the teaching culture, these teachers must continue to work with peers in the long term, such as by going to professional development meetings and conferences [25]. In particular, there is the need to create a learning community where beginning physical education teachers can participate and create an external network to the school, which then may enable the proper operation of cooperative and reflective professional development activities and systematic community-based practice activities [1].

Furthermore, the studies by Neil [51], Hanson-Smith [52], Lee and Yoo [23], Brooks and DinanThompson [53], and Cho et al. [25] argue that in-service teacher education should be strengthened. In particular, systematic support should be provided to change the culture of the schools to which beginning physical education teachers belong, and this support should shift from being work-oriented to class-oriented [54]. Brooks and DinanThompson [53] further argue that teachers’ administrative work should be reduced to create an atmosphere that enables them to spend more time and effort on enhancing their classes. Hence, these studies emphasize that teachers should strive to develop expertise, as well as that if the school culture in which teachers are involved does not change together with teachers, their efforts are likely to result only in temporary changes; then, teachers are likely to continue to be dependent on the work of a few outstanding teachers, like they currently do, and not be able to demonstrate sufficient collective capabilities by themselves.

Finally, the studies by Wood and Rhoades [55] and Cho et al. [25] argue that a lot of help should be provided to beginning physical education teachers to improve their expertise. On the topic of improvement, You et al. [1] describe three different growth types for beginning physical education teachers: potential, passive, and active. These authors then reported that the process through which beginning physical education teachers develop their expertise and adapt to the school varies at the individual level. This implies the need to support the development of beginning physical education teachers’ expertise using various participation and adaptation strategies, rather than following the existing organizational atmosphere, teaching methods, and work processing procedures. Since beginning physical education teachers are at a time in their careers when their passion for classes is likely to still be very high, our results and prior research point to the need for developing support strategies tailored for these professionals that enable them to focus on professional development during this initial period. Accordingly, the findings in this study should be used as basic data by invested stakeholders to find interventions that may help reduce the trial-and-error processes which these beginning teachers often experience and maximize their ability to effectively adapt to their jobs. This may in turn enable beginning physical education teachers to deliver proper guidance to their students.

### 4.3. Limitations and Suggestions for Future Research

First, this study was based on quantitative research, which means that we could not examine the stories, meanings, and contexts of the beginning physical education teachers in our sample. Future mixed-method studies are warranted to analyze more in-depth the difficulties experienced and efforts made by beginning physical education teachers during this initial period of their careers.

Second, the effect of occupational socialization of beginning physical education teachers on health perception education in the context of the COVID-19 pandemic situation was verified. As this study explored the factor structure of occupational socialization of beginning physical education teachers, various analyses should be conducted in future studies, such as identifying various variables and deriving results.

Third, sampling was rather difficult because the size of the targeted population was not large. Future studies should expand the sample to beginning physical education teachers from other countries and to make cross-country comparisons of physical education class contents, and of the expectations for the role of beginning physical education teachers.

## 5. Conclusions

In this study, we explored the factor structure of the occupational socialization of beginning physical education teachers scale and verified its relationship with health perception education. First, it was found that the scale has six subscales: role recognition, past physical education class experience, pre-service teacher education, organizational atmosphere, fellow physical education teachers, and sports facility. Second, occupational socialization of beginning physical education teachers had a partially positive effect on health perception education. These results suggested the need to identify the difficulties experienced by beginning physical education teachers during the COVID-19 pandemic and support them in their adaptation to the school environment to minimize the trial-and-error processes which they often experience when educating students.

## Figures and Tables

**Table 1:** Demographic characteristics of participants.

Variables	Classification	*n*	%
Sex	Male	183	71.2
	Female	74	28.8
School level	Middle school	166	64.6
	High school	91	35.4
Working years	Under 1 year	91	35.4
	1–2 year(s)	85	33.1
	Over 2–3 years	81	31.5
Total		257	100

**Table 2:** The results of exploratory factor analysis.

Items	Factor						Communality
	1	2	3	4	5	6	
Occupational socialization 16	0.865	0.195	0.143	0.120	0.129	0.144	0.859
Occupational socialization 17	0.851	0.203	0.181	0.104	0.227	0.064	0.864
Occupational socialization 18	0.840	0.237	0.208	0.182	0.119	0.091	0.860
Occupational socialization 19	0.676	0.304	0.318	0.217	0.212	0.064	0.747
Occupational socialization 20	0.522	0.473	0.221	0.211	0.207	0.101	0.643
Occupational socialization 22	0.106	0.798	0.079	0.035	0.279	0.189	0.769
Occupational socialization 23	0.165	0.755	0.077	0.226	0.259	0.134	0.739
Occupational socialization 25	0.187	0.748	0.071	0.086	0.226	0.091	0.667
Occupational socialization 21	0.257	0.732	0.168	0.191	0.073	0.098	0.681
Occupational socialization 24	0.395	0.635	0.224	0.198	−0.007	−0.039	0.650
Occupational socialization 6	0.243	0.147	0.803	0.166	0.141	0.161	0.798
Occupational socialization 8	0.183	0.136	0.780	0.118	0.150	0.219	0.744
Occupational socialization 7	0.271	0.101	0.767	0.218	−0.008	−0.049	0.722
Occupational socialization 9	0.167	0.156	0.526	−0.018	0.207	0.504	0.625
Occupational socialization 13	0.126	0.254	0.036	0.773	0.017	0.100	0.690
Occupational socialization 14	0.152	0.083	0.239	0.765	0.136	0.046	0.693
Occupational socialization 12	0.116	0.197	−0.052	0.700	0.034	0.371	0.684
Occupational socialization 15	0.134	0.047	0.190	0.602	0.151	0.126	0.458
Occupational socialization 4	0.117	0.195	−0.055	0.036	0.802	0.112	0.712
Occupational socialization 3	0.160	0.195	0.159	0.061	0.747	0.206	0.693
Occupational socialization 2	0.296	0.184	0.151	0.192	0.735	0.013	0.721
Occupational socialization 1	0.104	0.133	0.213	0.376	0.463	−0.067	0.434
Occupational socialization 5	0.014	0.168	0.347	0.019	0.381	0.259	0.362
Occupational socialization 11	0.093	0.132	0.110	0.230	0.120	0.840	0.811
Occupational socialization 10	0.107	0.132	0.183	0.230	0.112	0.838	0.830
Eigenvalue	9.603	1.960	1.850	1.648	1.292	1.101	-
Variance (%)	38.414	7.842	7.401	6.593	5.167	4.403	-
Accumulation variance (%)	38.414	46.256	53.657	60.250	65.417	69.820	-

**Table 3:** Confirmatory factor analysis results for the goodness of fit.

Index	Initial Goodness of Fit	Final Goodness of Fit
Chi-square/degree of freedom	2.772	2.38
Root mean square residual	0.037	0.032
Incremental fit index	0.889	0.924
Comparative fit index	0.888	0.923
Root mean square error of approximation	0.083	0.073

**Table 4:** Results of the confirmatory factor analysis.

Sub-Variables			Standardized Coefficient	Non-Standardized Coefficient	Standard Error	Critical Ratio	*p*	Average Variance Extracted
Role recognition	→	Occupational socialization 18	0.783	0.178	-	-	-	0.993
	→	Occupational socialization 19	0.910	0.077	0.013	5.681	<0.001 ***	
	→	Occupational socialization 20	0.832	0.112	0.013	8.554	<0.001 ***	
Past physical education class experience	→	Occupational socialization 21	0.752	0.279	-	-	-	0.992
	→	Occupational socialization 22	0.682	0.226	0.022	10.16	<0.001 ***	
	→	Occupational socialization 23	0.828	0.203	0.024	8.384	<0.001 ***	
	→	Occupational socialization 24	0.801	0.212	0.024	8.912	<0.001 ***	
	→	Occupational socialization 25	0.766	0.202	0.022	9.407	<0.001 ***	
Pre-service teacher education	→	Occupational socialization 6	0.883	0.084	-	-	-	0.991
	→	Occupational socialization 7	0.770	0.161	0.018	8.845	<0.001 ***	
	→	Occupational socialization 8	0.785	0.165	0.019	8.549	<0.001 ***	
Organizational atmosphere	→	Occupational socialization 12	0.571	0.420	-	-	-	0.982
	→	Occupational socialization 13	0.737	0.303	0.036	8.382	<0.001 ***	
	→	Occupational socialization 14	0.759	0.235	0.03	7.969	<0.001 ***	
	→	Occupational socialization 15	0.717	0.297	0.034	8.716	<0.001 ***	
Fellow physical education teacher	→	Occupational socialization 1	0.621	0.408	-	-	-	0.976
	→	Occupational socialization 2	0.914	0.084	0.029	2.842	<0.001 ***	
	→	Occupational socialization 3	0.641	0.454	0.047	9.74	<0.001 ***	
Sports facility	→	Occupational socialization 10	0.865	0.168	-	-	-	0.975
	→	Occupational socialization 11	0.909	0.119	0.042	2.817	<0.001 ***	

*** *p* < 0.001.

**Table 5:** Verification of discriminant validity.

Variables	Correlation between the Constructs						Average Variance Extracted
	Role Recognition	Past Physical Education Class Experience	Pre-Service Teacher Education	Organizational Atmosphere	Fellow Physical Education Teacher	Sports Facility	
Role recognition	1.000	-	-	-	-	-	0.979
Past physical education class experience	0.635 ***	1.000	-	-	-	-	0.961
Pre-service teacher education	0.572 ***	0.436 ***	1.000	-	-	-	0.975
Organizational atmosphere	0.458 ***	0.439 ***	0.400 ***	1.000	-	-	0.933
Fellow physical education teacher	0.514 ***	0.522 ***	0.420 ***	0.373 ***	1.000	-	0.933
Sports facility	0.332 ***	0.360 ***	0.463 ***	0.438 ***	0.351 ***	1.000	0.951

*** *p* < 0.001, tested by correlation analysis.

**Table 6:** Descriptive statistics are based on a five-point Likert response scale and scale reliability.

Variables		Mean	Standard Deviation	Skewness	Kurtosis	Cronbach’s α
Occupational socialization of beginning physical education teachers scale	Role recognition	4.35	0.56	−0.44	−0.45	0.923
	Past physical education class experience	4.10	0.61	−0.13	−0.37	0.875
	Pre-service teacher education	3.89	0.57	−0.30	0.68	0.820
	Organizational atmosphere	3.69	0.61	0.07	0.27	0.786
	Fellow physical education teacher	4.00	0.64	−0.61	0.38	0.774
	Sports facility	3.41	0.78	−0.03	−0.19	0.880

**Table 7:** The effect of occupational socialization of beginning physical education teachers on health perception education.

Dependent Variable	Independent Variable	Standard Error	β	*t*	*p*	Statistics
Health perception education	Constant	0.123	-	15.892	<0.001 ***	R = 0.580, R^2^ = 0.336, Adjusted R^2^ = 0.329, F = 47.279 *p* < 0.001 ***
	Role recognition	0.039	0.178	3.412	0.001 **	
	Past physical education class experience	0.036	0.088	1.704	0.089	
	Pre-service teacher education	0.028	0.184	5.004	<0.001 ***	
	Organizational atmosphere	0.038	0.054	1.027	0.305	
	Fellow physical education teacher	0.038	0.247	4.608	<0.001 ***	
	Sports facility	0.032	0.011	0.247	0.805	

** *p* < 0.01; *** *p* < 0.001, tested by multiple regression analysis.

## Data Availability

The data presented in this study are available upon request from the authors. The data are not publicly available owing to privacy and ethical restrictions.
